# Co-producing adult attention-deficit/hyperactivity disorder (ADHD) care in South Africa: A patient-centred framework for agency, trust, satisfaction and continuity

**DOI:** 10.4102/sajpsychiatry.v32i0.2618

**Published:** 2026-05-18

**Authors:** Dimpho Ditsebe, Renata Schoeman

**Affiliations:** 1 Stellenbosch Business School, Faculty of Management and Business Sciences, Stellenbosch University, Bellville, South Africa

**Keywords:** adult ADHD, co-production, patient involvement, agency, continuity of care, relational care

## Abstract

**Background:**

Despite advances in diagnostics and pharmacological interventions, adults with attention-deficit/hyperactivity disorder (ADHD) experience poor mental health outcomes in South Africa. Patient involvement in psychiatric care remains inconsistent, and co-production has yet to be explored for improved continuity, adherence, and satisfaction.

**Aim:**

This study explored how adults with ADHD perceive their involvement in psychiatric care to inform co-production practices for clinicians.

**Setting:**

Participants were recruited via the South African Society of Psychiatrists (SASOP) ADHD Special Interest Group (SIG).

**Methods:**

This qualitative study explored how adults with ADHD perceive their involvement in psychiatric care. Thirteen adults formally diagnosed with ADHD were purposively sampled. Semi-structured interviews, guided by the SERVPERF model, were conducted online and analysed thematically using ATLAS.ti (version 24.2.1 [32295]; ATLAS.ti Scientific Software Development GmbH, Berlin, Germany).

**Results:**

The proposed patient-centred care framework is based on four identified themes: (1) patient agency enhances engagement when decision-making is shared and treatment options are clearly explained; (2) trust in provider expertise and empathy influence adherence and comfort with treatment plans; (3) satisfaction increases with personalised, responsive care, particularly during medication adjustments and follow-up; and (4) continuity of care and timely access foster sustained engagement, while inconsistent communication leads to frustration and dropout. Together, these dynamics form a relational framework for the co-production of care.

**Conclusion:**

Patient involvement in ADHD care is relational and influenced by trust, effective communication, and continuity of care. Where co-production was present, patients experienced improved outcomes.

**Contribution:**

This study emphasises integrating co-production into psychiatric practice through structured involvement strategies, improved continuity, and relational training for healthcare providers.

## Introduction

Co-production, a model that actively involves patients as partners in designing, delivering and evaluating their treatment, has gained traction across global health systems as a relational and implementation strategy. It positions patients as active design partners in the delivery and evaluation of care, improving satisfaction, adherence and long-term outcomes.^1,2,3^ For psychiatrists treating adult attention-deficit/hyperactivity disorder (ADHD), the paradox is clear: Even with accurate diagnoses and effective medications, meaningful, sustained outcomes often remain elusive.^4,5,6,7^ Despite notable advances in psychiatric diagnostics and pharmacological interventions, mental health outcomes in adults with ADHD remain persistently poor.^8,9,10^ Increasingly, international mental health discourse recognises that treatment success depends not only on clinical efficacy but also on how care is experienced, particularly the extent to which patients feel heard, involved, and respected.^11,12,13^

In South Africa, only about 1.09% of adults in the private healthcare sector have received a formal ADHD diagnosis,^5,6,7^ a figure widely considered to be a significant underestimation. The country’s dual healthcare system is characterised by marked inequities in access to specialist psychiatric services, particularly in the public sector.^14^ Access to a diagnosis of ADHD and treatment is often constrained by language, geographic, and financial barriers.^15^ This suggests a large population of undiagnosed adults, particularly in the public sector and underserved communities.^16^ Despite this diagnostic gap, people in the private sector remain largely absent from the design of mental health services.^8,17,18^ Psychiatric care continues to operate in silos, with clinical decisions primarily shaped by provider perspectives, rather than being co-informed by patients’ insights.^9,19^ This approach poses risks, particularly in chronic mental health conditions where engagement and continuity of care are essential for long-term outcomes.^20,21^ These challenges, however, are not merely technical; patterns of treatment disengagement, reports of feeling unheard, and strained therapeutic relationships suggest that they may extend beyond the technical.^8,18,22^ They hint at a deeper relational gap in how care is co-constructed between clinicians and patients. As highlighted by the Life Esidimeni tragedy, where 144 psychiatric patients died after being transferred from licensed care facilities to inadequately resourced non-profit organisations as part of a cost-cutting project, the consequences of excluding patient voices from mental health planning and delivery can be severe.^17,23,24^

To conceptualise this relational component more holistically, Bronfenbrenner’s ecological systems theory offers a valuable framework. It illustrates how individual functioning is shaped through interconnected systems, ranging from the immediate doctor-patient relationship (microsystem) to family and community networks (mesosystem) and broader factors such as healthcare design and clinician workload (exosystem).^25,26,27^ At the macrosystem level, cultural norms, workplace demands, and healthcare policies influence how patients experience care.^21,28^ Failure to consider these layers can lead to fragmented treatment and missed opportunities for co-production.^29^

Psychiatrists and other mental healthcare workers are well-positioned to use patients’ insights and perspectives to personalise care, improve engagement, and address barriers to continuity of care.^30,31,32^ Understanding the lived experience of adults with ADHD, including what they value in care, what they struggle with, and how they define success, is essential for developing responsive, value-based healthcare interventions.^33,34,35^ However, as confirmed by McCalman et al., specifics of patient involvement and the factors influencing its effectiveness remain largely unexplored.^23^

Although co-production has become a core tenet in high-income countries, its empirical application within South African psychiatric services remains underexplored, particularly in adult ADHD care.^16,36^ This study addresses this gap and explores how adults with ADHD in South Africa perceive their involvement in care and what clinicians can learn from their experiences to improve adherence and outcomes. The insights on embedding co-production principles into psychiatric practice could challenge traditional, top-down models of care in favour of more responsive, collaborative, patient-informed approaches and interventions. By highlighting how co-production can operate as a relational and implementation practice, this study contributes to the global conversation on reconfiguring mental healthcare toward more responsive, collaborative, and sustainable models.

## Research methods and design

### Study design

This qualitative study explored how adults with ADHD perceive their involvement in care.

Qualitative research was selected for its ability to capture nuanced, context-rich accounts from these adults, reflecting their lived experiences and relational dynamics in mental health practices.^11,37,38,39^ The study was grounded in a constructivist paradigm, recognising that meaning emerges through the interaction between the researcher and the participant.

### Sampling and study population

The study population consisted of 13 (*n* = 13) adults, primarily private-sector patients, who had been formally diagnosed with ADHD by a psychiatrist and were able to access psychiatric care, reflecting a relatively resourced and well-connected sample. The sample comprised seven males and six females, ranging in age from 23 years to 49 years, with a duration since diagnosis ranging from recently diagnosed (4 months) to 14 years. Adults were chosen as the study’s focus group because they pose fewer ethical complexities than minors, who require additional protections in research contexts.^40,41,42,43^ Purposive sampling was used to recruit participants through the South African Society of Psychiatrists (SASOP) Special Interest Group (SIG) for ADHD, utilising a WhatsApp-based group and the experts’ network connections.^22,44^ Five psychiatrists were initially invited to refer approximately three participants each. However, due to challenges and a slow response rate, recruitment was extended until data saturation was reached.^38^

Purposive sampling was employed to ensure participants had a formal psychiatric diagnosis and direct treatment experience, which was essential to exploring relational dynamics in ADHD care. While participants were required to have a formal ADHD diagnosis confirmed, the study did not specifically screen for or control for the presence of psychiatric comorbidities.^45^

### Data collection

Data were collected over a 4-week period through individual semi-structured interviews conducted via Microsoft Teams. Interviews were conducted in English, which was the preferred language of all participants. This modality accommodated participants’ varying geographic locations and time constraints.^8,23^ Audio-only interviews were undertaken to minimise visual bias. Interviews lasted between 45 min and 60 min and were recorded with participants’ consent to enable transcription and analysis.

Interview questions were guided by the SERVPERF model, which evaluates service quality based on participant perceptions.^46,47,48^ Questions were developed to explore participants’ experiences of ADHD treatment, their involvement in care decisions, and perceptions of co-production.^6,49^ The interview guide was reviewed by a senior researcher with expertise in qualitative methods and psychiatric clinical experience to ensure clarity, readability, and contextual appropriateness. Given the nature of the sample, adults with ADHD, efforts were made to minimise participant burden, and full participation in this validation step was not pursued to avoid overwhelming respondents with paperwork and follow-up tasks.

### Data analysis

The data were examined using thematic analysis. Transcripts were reviewed and manually coded to identify features relevant to the research questions. The codes were then organised into preliminary themes, which were iteratively refined to ensure internal coherence and alignment with the study objectives.^11,50^ Clear definitions were developed for each of the final themes. ATLAS.ti (version 24.2.1 [32295]; ATLAS.ti Scientific Software Development GmbH, Berlin, Germany) was used to support coding, theme development, and mapping of relationships within the data. This facilitated structured analysis, ensured transparency in coding decisions, and strengthened the study’s rigour.^38,51^

### Ethical considerations

Ethical approval for this study was obtained from the Stellenbosch University Health Research Ethics Committee (HREC) (Project ID 31419 and HREC Reference Number S24/07/183). The study adhered to the ethical standards set out in the Declaration of Helsinki (1964) and its subsequent amendments. Written informed consent was obtained from all participants prior to the interviews.

## Results

The diversity in participant age, professional background and duration since diagnosis of ADHD contributes to a rich dataset, allowing for exploration of how these factors may influence patient involvement in co-production within mental healthcare. While the study ensured diversity, it did not collect detailed information on psychiatric comorbidities. This is relevant as ADHD frequently co-occurs with other conditions such as mood disorders, anxiety disorders, substance use disorders, and personality disorders, which may influence patient experiences of care and treatment engagement.

Notably, although treatment adherence was not an inclusion criterion, two participants had disengaged or dropped out of care. Their perspectives contributed valuable insights into the challenges of continuity of care and added depth to the emerging conversation on service co-production in mental healthcare.

As outlined in Table 1, this demographic variation provides context for understanding the personalised nature of patient care and patients’ involvement across different settings.

**TABLE 1:** Participants’ demographics.

Participant ID	Age (years)	Gender	Occupation	Duration since diagnosis (years)
P1	44	Female	Executive	9.00
P2	32	Male	Industrial Engineer	12.00
P3	49	Female	Accountant	0.50
P4	48	Male	Engineer	13.00
P5	49	Male	Programmer	14.00
P6	42	Male	Medical Doctor	14.00
P7	25	Female	Registered Counsellor	4.00
P8	38	Female	Legal Professional	5.00
P9	23	Female	Student	2.00
P10	23	Male	Architect	6.00
P11	40	Male	Medical Doctor	0.33
P12	30	Male	Intern Psychometrist	6.00
P13	32	Female	Student	2.00

ID, Identity.

The final code list consisted of 45 codes, representing 351 comments. The codes were then grouped into 11 subthemes, from which four key themes emerged: Patient agency and involvement, Trust in healthcare providers, Satisfaction with care and outcomes, and accessibility and continuity of care. These themes represent the participants’ reflections and offer insight into the relational dynamics and structural barriers shaping their experiences of care. Results will be reported as participant: quote; for example, (P4:Q3) refers to participant 4, quote 3.

### Patient agency and involvement

Participants involved in decision-making reported greater control and accountability over their treatment. One patient mentioned:

‘It just made me feel like she’s also human instead of her profession, so it was easier to connect with her and feel safer with her because I felt like she would recognise me for me instead of me for my diagnosis.’ (P9, Q1)

This sense of agency was particularly strengthened when providers offered transparent communication, relational engagement, and tailored support. These relational interactions empowered patients to not only understand their care but to take ownership of it:

‘She was very accommodating. She explained to me the effects of the medication, what’s new, what can change, and that’s also where she can change my antidepressants to something else that will work better with my new ADHD medicine.’ (P3, Q6)

Participants consistently indicated that such empowerment increased their commitment to care. One noted how provider support directly shaped her ability to follow through with the treatment plan:

‘I was extremely confident, which then meant that the whole care plan, everything, I was much more likely to actually follow through with her suggestions.’ (P8, Q5)

However, this unstructured autonomy, while well-intentioned, could undermine patient confidence and threaten long-term adherence, particularly when patients were unsure how to weigh their options:

‘I think professional, the practitioners will give you options in terms of the medication and what, the side effects of each and what they recommend, but there’s always, there’s never a, you know, it’s almost. They give you the confidence, but hey, you know, it’s up to you, type of vibe.’ (P1, Q11)

In contrast, others appreciated when information was contextualised through dialogue:

‘She explained all of that to me, which was very nice.’ (P3, Q6)

This type of engagement helped reduce emotional fatigue and enhanced adherence, as patients felt informed and accompanied in their care journey.

Conversely, a lack of early guidance delayed treatment and left some feeling unsupported at critical points:

‘When we eventually got the care and … I was diagnosed with ADHD and then put onto the correct medication; it was life-changing.’ (P5, Q7)

This highlights how delays in diagnosis and relational disconnect can erode patient empowerment and jeopardise care continuity. Personalised care was consistently identified as a driver of engagement:

‘So I very much feel like when I go sit in his office, I as the person is in the centre, and then everything else is secondary to that.’ (P6, Q12)

### Trust in healthcare

Trust in the provider’s knowledge, consistency, and empathy influenced patients’ engagement with their care. This trust was clear when a patient said:

‘They understood the situation well … I didn’t have any reason to doubt their diagnosis.’ (P2, Q16)

Another mentioned that they feel more confident in their psychiatrist’s knowledge and skills than their own. These responses suggest that trust was based not only on how well providers understood ADHD but also on how effectively they communicated this expertise to the patient.

Empathy and communication from healthcare providers are critical in building trust, and patients who received it said:

‘She was very empathetic and very kind and made me kinder and more empathetic to myself.’ (P9, Q20)

Participants reported that providers who took the time to listen to them and clearly explained treatment options fostered a greater sense of security and comfort. Conversely, participants who felt their providers were not empathetic or communicative were less likely to trust the treatment process. One described an experience where a lack of communication led to uncertainty:

‘I’ve never experienced a sense of empathy at the clinics. Specifically, now related to my ADHD treatment plan.’ (P12, Q32)

These responses suggest that empathy and effective communication are essential for building trust.

### Satisfaction with care and outcomes

Participants who experienced involvement and emotional support reported greater satisfaction and treatment adherence. Satisfaction is influenced by how well symptoms are managed, how well the treatment aligns with patients’ personal goals and lifestyles, and the extent of their level of involvement in creating treatment plans. Participants involved in decisions and received tailored care described higher satisfaction:

‘It was sort of life-changing … be able to showcase my abilities … less turbulent and get a greater understanding of myself and compassion.’ (P8, Q30)

This quote illustrates that patients felt most satisfied when providers adapted their treatment plans to the patient’s individual experiences rather than relying on generic treatment protocols. Participants who were actively involved in selecting their medications and treatment strategies reported greater satisfaction, reflected in the example below:

‘I was happy with the results because I felt like the plan was tailored to me, not just based on what works for most people.’ (P7, Q34)

However, participants also mentioned instances when medication adjustments were necessary due to inefficacy or side effects, reinforcing the need for continuous involvement in decision-making, as one noted:

‘Brand 1 didn’t work for me … but Brand 2 worked without suppressing my appetite. That change improved everything.’ (P1, Q35)

Participants also highlighted the value of consistent follow-up and communication:

‘I was always treated as a person, a patient first who happened to have a bit of extra specialised knowledge, and he would always check in with me.’ (P6, Q38)

This shows how continuous support and consistent communication from healthcare providers contributed to building trust and overall satisfaction with care.

### Accessibility and continuity of care

Access to consistent providers and continuity of care emerged as critical. Participants consistently highlighted the importance of reaching their healthcare providers promptly. When communication was timely, patients felt reassured and engaged in their treatment:

‘I think the availability to say, “contact me at any time necessary.” It’s more like, send me an email if you have any concerns or questions. I think that’s very reassuring.’ (P10, Q39)

This sense of accessibility helped participants feel empowered to raise concerns and seek support, which in turn reinforced their adherence to treatment plans.

Participants reported feeling more confident and secure when they experienced continuity from the same provider. This consistency helped build trust and stability in the treatment journey, encouraging patients to remain engaged and motivated. A patient explained how continuity positively influenced their experience:

‘I’ve never felt dismissed or that my concerns were dismissed …’ (P6, Q42)

Inconsistent care, by contrast, led to dissatisfaction:

‘There was one time … but most of the time there’s a long delay.’ (P4, Q44)

Overall continuity supported relationship-building and trust, while disruptions undermined confidence in care systems.

## Discussion

**FIGURE 1:**
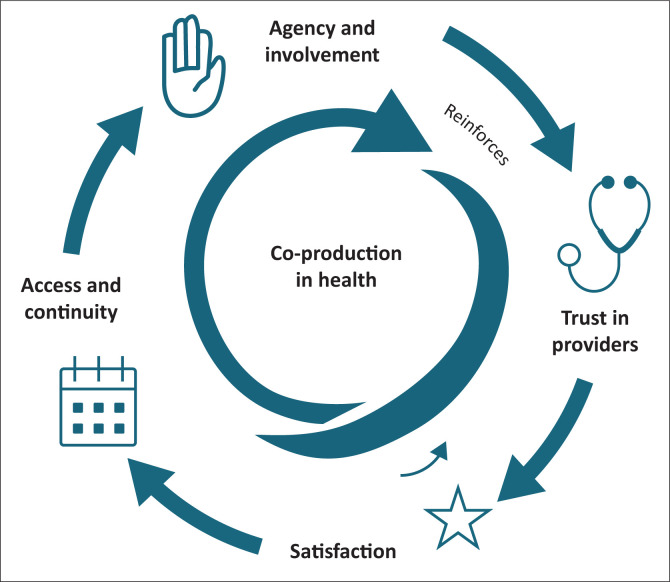
A patient-centred care framework for co-producing adult attention-deficit/hyperactivity disorder care.

Drawing on the results, Figure 1 presents a patient-centred care framework where these four interrelated themes, namely patient agency, provider trust, satisfaction with care, and continuity, form a relational cycle that underpins co-production in psychiatric care.

Based on the findings, the patient-centred care framework aligns with prior research, highlighting the importance of patient agency in promoting collaborative clinical decision-making and therapeutic partnership.^34^ For some participants, agency was a defining feature of their treatment journey. Others described limited engagement with care decisions, often shaped by the quality of communication with their psychiatrist. These reflections illustrate that patient involvement is neither uniform nor guaranteed.

Notably, trust in provider expertise influenced perceived involvement, aligning with Higgins et al.’s suggestion that trust is foundational to collaborative care.^33,52,53^ Trust, a core element in mental healthcare, was influenced by how providers acknowledged patients’ lived experiences, echoing Sunkel and Sartor’s emphasis on the need for relational approaches in mental health.^13,19,22^ Therefore, trust emerged as a key factor in determining engagement.

Participants who described their providers as approachable and consistent were more likely to report active participation, while those who experienced poor communication described disengagement. This finding aligns with Charles et al. who emphasise the relationship between agency and collaborative satisfaction.^31,32,33,34^

It was noted that increased involvement was linked to greater satisfaction, empowerment, and adherence. Participants highlighted how co-developed care plans enhanced their sense of control and autonomy. At the same time, others noted that involvement helped them better understand and commit to treatment. These experiences reflect the benefits of shared decision-making.^11,54^ Empathy and support structures also played a crucial role, as Ong et al. argue that empathetic communication fosters engagement and enhances treatment outcomes.^31,49,55^ Participants who experienced co-production described higher satisfaction and improved outcomes, particularly when supported by increased accessibility and consistent follow-up. These insights align with evidence that co-production and continuity of care improve patient outcomes and satisfaction.^9,30,56^

Similar patterns have been reported in high-income contexts such as Canada, the UK, and the Netherlands, where shared decision-making and relational continuity are central to national mental health policies. In these settings, co-production has been linked to patient satisfaction, as well as reduced service utilisation and improved cost-effectiveness, underscoring its value beyond individual engagement.^2,32,57^ Accessibility and continuity of care are influenced not only by clinician availability but also by the organisational processes that support patient access to services. In many healthcare settings, reception and administrative staff represent the first point of contact for patients and therefore play an important role in shaping patients’ experiences of access and continuity.^58^ Administrative processes related to appointment scheduling, prescription renewals and communication with clinicians can either facilitate timely engagement with care or contribute to delays and frustration for patients managing chronic conditions such as ADHD.

However, unlike in contexts where co-production is increasingly institutionalised, South Africa lacks formal structures and policy frameworks that embed patient involvement in psychiatric service delivery. This disparity highlights a pressing implementation gap, especially in areas such as adult ADHD, which remains underdiagnosed and poorly integrated into public mental health strategies.^13,59^

By positioning co-production as both a relational and system-level intervention, this study contributes to global discussions on adapting participatory care models to under-resourced settings.^60^ The cyclic interplay between involvement, trust, satisfaction, and continuity aligns with the ecological systems framework,^4,27^ suggesting that interventions should address individual and systemic levels to sustain engagement.

### Strengths and limitations

A key strength of the study is the depth of insight provided through patient narratives, which enabled the identification of interconnected relational themes. The inclusion of underrepresented voices in ADHD care, particularly those who have dropped out of treatment, added value to the emerging discourse on service co-production. However, several limitations should be acknowledged. Firstly, the study did not systematically collect information on psychiatric comorbidities. Adults with ADHD frequently experience co-occurring conditions such as mood and anxiety disorders, which may influence perceptions of treatment and engagement with care. Secondly, the relatively small sample size (*n* = 13), while appropriate for qualitative inquiry and sufficient to achieve thematic saturation, limits the transferability of the findings to broader ADHD populations. Thirdly, the study relied on retrospective self-reports, which may introduce recall bias. Finally, findings must be interpreted within the context of a relatively resourced, private-sector population. Experiences of patient agency, trust and continuity may differ substantially in public sector settings characterised by high patient loads, language discordance and limited specialist access.

### Implications or recommendations

Recognising that patient involvement is dynamic and context-sensitive, the study’s findings offer critical implications for mental health practice, particularly in shaping training, service design, and relational care strategies. Findings support integrating relational care training into psychiatric education and revising care models to assess patient involvement routinely.

Health systems should incorporate not only patient-related outcome measures but also patient-reported experience measures as core quality indicators. Future research should investigate how these relational cycles impact clinical outcomes and explore provider perceptions of agency-building strategies, particularly across diverse socioeconomic contexts.

These implications underscore the study’s core message: enhancing outcomes in adult ADHD care necessitates a systemic commitment to relational co-production, not just clinical precision.

## Conclusion

This study contributes to a growing body of literature that moves beyond clinical efficacy to explore the relational and structural dimensions of care. The findings demonstrate that a dynamic interplay of agency, trust, satisfaction, and continuity shapes patient involvement in adult ADHD care. Qualitative methods enabled a deep exploration of lived experience, revealing how small relational moments, such as empathetic communication or consistent follow-up, can influence long-term engagement. The SERVPERF-informed interview approach elicited perceptions of care quality in a psychiatric setting.

For practitioners, this study emphasises the importance of fostering trust, encouraging patient agency, and ensuring continuity in provider relationships. For health system managers, it underlines the value of incorporating patient-reported experiences into quality improvement strategies. For researchers, it signals the need to investigate further how relational cycles operate across diverse clinical contexts. Theoretically, the findings affirm the utility of Bronfenbrenner’s ecological systems model as a lens for understanding patient engagement. Future research should test the framework in under-resourced or public sector contexts.

Empirically, they offer patient-informed evidence to guide the integration of co-production principles into psychiatric care. Ultimately, advancing outcomes in adult ADHD care requires both clinical excellence and a deliberate, relational approach, care that listens, evolves and remains consistently engaged.
